# Biochemical Similarities and Differences between the Catalytic [4Fe-4S] Cluster Containing Fumarases FumA and FumB from *Escherichia coli*

**DOI:** 10.1371/journal.pone.0055549

**Published:** 2013-02-06

**Authors:** Barbara M. A. van Vugt-Lussenburg, Laura van der Weel, Wilfred R. Hagen, Peter-Leon Hagedoorn

**Affiliations:** Department of Biotechnology, Delft University of Technology, Delft, The Netherlands; Institute of Molecular Genetics IMG-CNR, Italy

## Abstract

**Background:**

The highly homologous [4Fe-4S] containing fumarases FumA and FumB, sharing 90% amino acid sequence identity, from *Escherichia coli* are differentially regulated, which suggests a difference in their physiological function. The ratio of FumB over FumA expression levels increases by one to two orders of magnitude upon change from aerobic to anaerobic growth conditions.

**Methodology/Principal Findings:**

To understand this difference in terms of structure-function relations, catalytic and thermodynamic properties were determined for the two enzymes obtained from homologous overexpression systems. FumA and FumB are essentially identical in their Michaelis-Menten kinetics of the reversible fumarate to L-malate conversion; however, FumB has a significantly greater catalytic efficiency for the conversion of D-tartrate to oxaloacetate consistent with the requirement of the *fumB* gene for growth on D-tartrate. Reduction potentials of the [4Fe-4S]^2+^ Lewis acid active centre were determined in mediated bulk titrations in the presence of added substrate and were found to be approximately −290 mV for both FumA and FumB.

**Conclusions/Significance:**

This study contradicts previously published claims that FumA and FumB exhibit different catalytic preferences for the natural substrates L-malate and fumarate. FumA and FumB differ significantly only in the catalytic efficiency for the conversion of D-tartrate, a supposedly non-natural substrate. The reduction potential of the substrate-bound [4Fe-4S] active centre is, contrary to previously reported values, close to the cellular redox potential.

## Introduction

**Figure 1:**
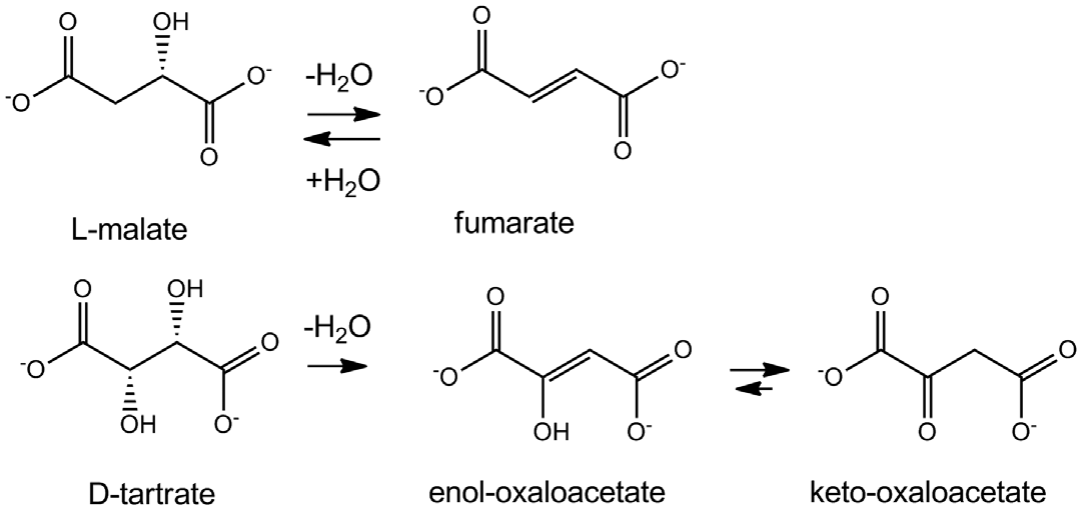
Reaction scheme of the L-malate dehydration (*top*) and the D-tartrate dehydration reactions (*bottom*) catalyzed by fumarase.

Fumarase, or fumarate hydratase (EC 4.2.1.2) plays a central role in the tricarboxylic acid (TCA) cycle. It catalyzes the reversible hydration of fumarate to L-malate, and also the dehydration of D-tartrate to oxaloacetate [1], [2] (Figure 1). *E. coli* possesses three fumarate hydratases. The class I fumarases FumA and FumB contain an oxygen-sensitive catalytic [4Fe-4S] cluster, which is readily oxidized to an inactive [3Fe-4S] cluster [3], [4]. One of the iron ions of the [4Fe-4S] cluster is not coordinated by an amino acid residue, and is available to participate in catalysis [4]. This iron acts as a Lewis acid to activate a hydroxyl, either from the substrate for elimination or from water for addition [5]. Class I fumarases are found in bacteria, predominantly enterobacteria and bacteriodetes such as *Salmonella* and *Klebsiella*. The iron-independent, oxygen-stable FumC belongs to class II [6] and is homologous to eukaryotic fumarases. More recently a third type of fumarase was found in the thermophilic bacterium *Pelotomaculum thermopropionicum* and hyperthermophilic archaeon *Pyrococcus furiosus*, which is heterodimeric and also relies on a [4Fe-4S] cluster as a catalytic centre [7], [8].

Although FumA and FumB share a high degree of sequence identity (90% amino acid sequence identity in *E. coli*), they are expressed under different physiological conditions. FumA has been identified as the major TCA cycle enzyme in *E. coli*, since it is expressed under aerobic conditions, and repressed by glucose and anaerobiosis [9]. FumB is mainly expressed during anaerobic growth [9]–[11] to the extent that the ratio of FumB over FumA expression levels can increase by up to a factor of 60 upon changing from aerobic to anaerobic growth conditions [9]. Recently the reduction in fumA transcription after an increase in growth rate has been found to be compensated by an increase in the half-life of the fumA mRNA causing the FumA protein levels to be unchanged [12]. The FumA and FumB protein levels under aerobic and anaerobic conditions have not been reported. The difference in regulation of these two enzymes suggests different physiological functions, and therefore different enzymatic properties. The aim of the current study is to find an explanation why *E. coli* possesses two enzymes with a high degree of homology, and why they are differentially regulated. To accomplish this, FumA and FumB were extensively characterized with respect to kinetic parameters, stability, and properties of the [4Fe-4S] clusters. FumA has already been characterized to a certain extent previously [3], [5], but for FumB few experimental data have been published [13].

## Results

### Enzyme Expression, Purification and Characterization

SDS PAGE analysis of *E. coli* cells containing pET15B-FumA/B after overnight induction showed efficient overexpression of FumA and FumB (Fig. S1). FumA has previously been purified as a homodimer [1], [3], [6]. Size exclusion chromatography showed that the molecular weight of native FumB, too, is circa 120 kDa, corresponding to a homodimer (not shown). EPR spectroscopy and activity measurements on lysed cells showed that the reactivated enzymes contained intact [4Fe-4S] clusters (Fig. S2). However, after purification on a nickel-sepharose column, only inactive enzyme containing [3Fe-4S] clusters was detected. The [3Fe-4S] cluster containing enzymes could be regenerated by incubation with Fe^2+^ and L-cysteine under anaerobic conditions, resulting in full restoration of activity. Iron concentration determination indicated that only 10–25% of the purified FumA and FumB contained a [3Fe-4S] cluster, the remainder being apoprotein.

### EPR Spectroscopy

**Figure 2:**
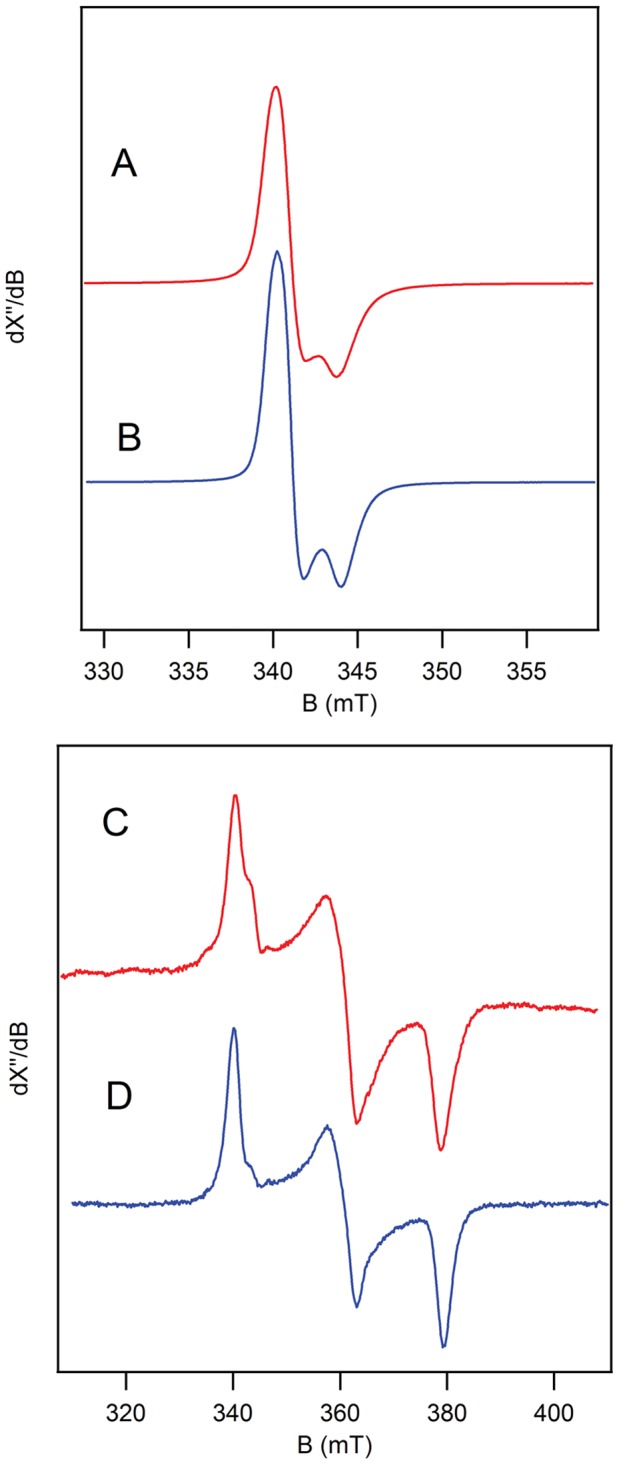
EPR spectra of FumA (red) and FumB (blue). a FumA [3Fe-4S]^1+^ clusters; as isolated, not regenerated. b FumB [3Fe-4S]^1+^ clusters; as isolated, not regenerated. c FumA [4Fe-4S]^1+^ clusters; regenerated, reduced and in the presence of 5 mM fumarate. d FumB [4Fe-4S]^1+^ clusters; regenerated, reduced and in the presence of 5 mM fumarate. The g-values are 2.032, 1.914 and 1.822 for FumA and 2.032, 1.916 and 1.821 for FumB. EPR parameters: microwave frequency a 9.630 GHz, b 9.631 GHz, c 9.407 GHz, d 9.631 GHz; microwave power a 8.0 mW, b 8.0 mW, c 20 mW, d 20 mW; modulation frequency 100 kHz; modulation amplitude a 0.63 mT, b 0.63 mT, c 1.25 mT, d 1.25 mT; temperature a 14.5 K, b 14.5 K, c 16K, d 14.5 K.

The EPR spectra of FumA and FumB ‘as isolated’ are very similar, showing only a subtle difference in shape (Figure 2A and 2B), indicating subtle differences in the micro-environment of the Fe-S cluster. The S = 1/2 spectra are characteristic for a [3Fe-4S]^+^ cluster [14]. Regeneration of the enzyme by the addition of Fe^2+^ and cysteine, followed by reduction with sodium dithionite in the presence of fumarate and/or L-malate results in a signal from the [4Fe-4S]^1+^ cluster, apparently with substrate and/or product bound. The signals of FumA and FumB are very similar (Figure 2C and 2D). The EPR spectra of FumA were similar to spectra published by Flint et al. [3].

### EPR Monitored Redox Titrations

**Figure 3:**
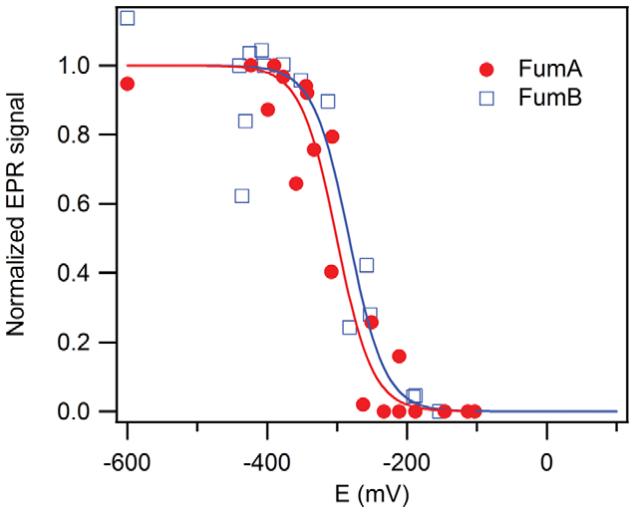
EPR-monitored redox titration curves of FumA (•, red) and FumB (□, blue). The two points at low, undefined potential represent samples reduced with excess dithionite (10 mM). The solid lines represents fits to the Nernst equation: 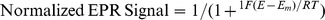. Fit parameters for FumA: E_m_ = −300±6 mV; and for FumB: E_m_ = −283±9 mV.

The midpoint potentials of FumA and FumB at pH 8 are −300±6 mV and −283±9 mV respectively (Figure 3). Given the experimental error in the redox titration (ca. 20 mV in the potential) we have to conclude that the [4Fe-4S]^2+/1+^ reduction potentials of FumA and FumB are not significantly different.

### Enzyme Activity

FumA and FumB catalyze the fumarase reaction in both directions with very similar kinetic parameters (Table 1, see Table S1 and Figure S3 for the original data). In contrast, the rate of D-tartrate dehydration by FumA was 5-fold lower than by FumB, while the *K_m_* values were similar for the two enzymes. The enzymes were not able to convert D-malate, L-tartrate or meso-tartrate (not shown). This substrate specificity is consistent with previously published results on FumA [5]. The catalytic efficiencies found here for FumA were very similar to previously published values by Flint [5]: k_cat_/K_m_ = 5·10^6^ s^−1^ M^−1^ for fumarate and 0.9·10^6^ s^−1^ M^−1^ for L-malate as compared to the values presented here: 4.5·10^6^ s^−1^ M^−1^ for fumarate and 1.4·10^6^ s^−1^ M^−1^ for L-malate.

**Table 1:** Kinetic parameters of fumarase and tartrate dehydratase activity of FumA and FumB^a^.

	L-malate			fumarate				D-tartrate		
	b *K_m_*	c *V_max_*	d *k_cat_/K_m_*	*K_m_*	*V_max_*	*k_cat_/K_m_*	e *K_eq_*	*K_m_*	*V_max_*	*k_cat_/K_m_*
FumA	0.7±0.1	(7.2±0.4)·10^2^	(1.0±0.2)⋅10^6^	0.46±0.08	(19±1)·10^2^	(4.2±0.8)⋅10^6^	4±1	0.8±0.3	2.3±0.3	(3±1)⋅10^3^
FumB	0.30±0.07	(4.9±0.3)·10^2^	(1.6±0.4)⋅10^6^	0.32±0.04	(14.3±0.6)·10^2^	(4.5±0.6)⋅10^6^	2.8±0.8	0.8±0.1	9.2±0.4	(12±2)⋅10^3^

a All values are corrected for Fe-S cluster content.

b *K_m_* in mM.

c *V_max_* in µmol product/minute/mg enzyme.

d *k_cat_/K_m_* in s^−1^ M^−1^.

e *K_eq_* is the equilibrium constant for the hydration of fumarate as calculated using the Haldane relationship: *K_eq_* = *(k_cat_/K_M_)_fumarate_/(k_cat_/K_M_)_L-malate_*.

### Enzyme Stability

**Figure 4:**
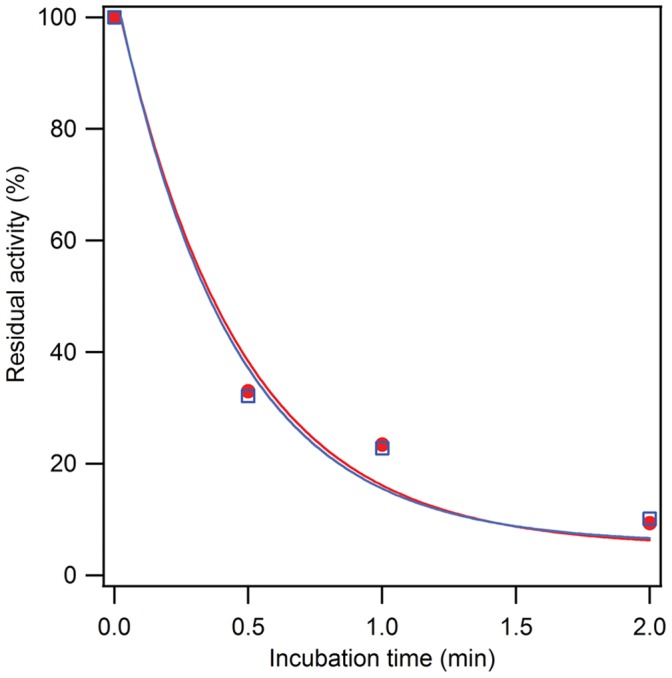
Oxygen sensitivity of FumA (•, red) and FumB (□, blue). Fumarase activity was measured after air oxidation for 0, 0.5, 1 or 2 minutes. The residual activity was plotted as a percentage of the initial activity. The solid lines represent fits to the following equation: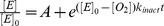. The fit parameters were as follows, for FumA: *A* = 5±6%; *k_inact_* = (1.8±0.5)·10^2^ M^−1^s^−1^, for FumB: *A* = 6±6%; *k_inact_* = (1.6±0.4)·10^2^ M^−1^s^−1^.

The oxygen sensitivities of FumA and FumB were very similar. For both enzymes exposure to air for 30 seconds resulted in a decrease in activity of ∼70%. After two minutes exposure to air, only 10% of the activity remained (Figure 4). The second order rate constants derived from this experiment were: 1.8±0.5·10^2^ M^−1^s^−1^ for FumA and 1.6±0.4·10^2^ M^−1^s^−1^ for FumB. Similar second order rate constants for the inactivation by oxygen of FumA and FumB have been reported previously to be 3·10^2^ M^−1^s^−1^ for FumA and 2·10^2^ M^−1^s^−1^ for FumB [13]. We did not find any significant difference in the rate of oxygen inactivation between FumA and FumB.

Upon photoreduction of the [4Fe-4S]^2+^ cluster with proflavin, the activity of FumA and FumB decreased to 0–3% of the initial activity (not shown). Re-oxidation resulted in restoration of the activity to 50–90%. Similar findings have been described previously for FumA only [3].

## Discussion

The aim of the current study was to find an explanation why *E. coli* possesses two differentially regulated fumarase paralogs. FumA expression is transcriptionally activated by Crp and inhibited by ArcA and Fnr [11], [12], [15], [16]. FumB expression is transcriptionally activated by Crp, ArcA, DcuR, Fnr and Fur, and inhibited by Fis and NarL [10], [16]. These regulatory effects can be summarized as a response to anaerobiosis, availability of dicarboxylates and availability of iron. Each of these enzymes must have some uniquely useful properties that are yet to be identified. The *fumB* gene is on a transcriptional unit with *dcuB*, which encodes a C4-dicarboxylate/succinate antiporter. The transcription of these genes together with the genes encoding fumarate reductase (frdABCD) is activated by C4-dicarboxylates, such as L-malate or L-tartrate, via the DcuS/DcuR two-component system. For instance in the metabolism of L-tartrate by *E. coli*, L-tartrate is converted to L-malate and subsequently dehydrated by FumB to fumarate, which then enters the fumarate respiration pathway [17].

**Figure 5:**
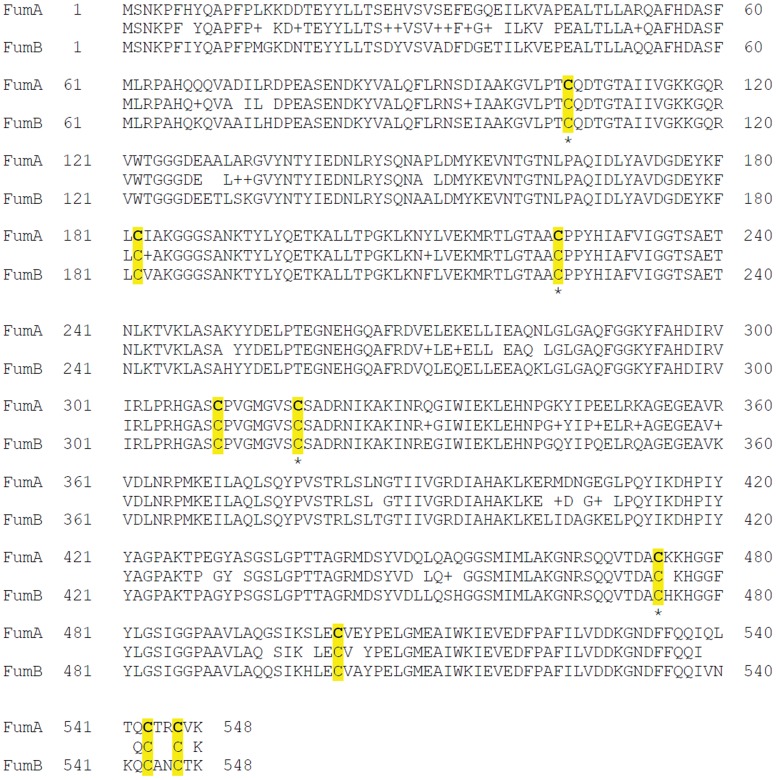
Amino acid sequence alignment of *E. coli* FumA and FumB. FumA and FumB share 90% sequence identity and 95% sequence similarity. *Cysteine residues strictly conserved in multiple sequence alignment, using the ‘Cobalt’ program, from the 500 homologs exhibiting >72% sequence identity with *E. coli* FumA.

The amino acid sequence similarity between FumA and FumB is very high, however, amino acid sequence alignment of 500 homologs with more than 70% sequence identity revealed that only four out of the nine cysteines are strictly conserved (Figure 5). Out of these four, C105, C224, C310 and C474, three are likely to be involved in the coordination of the active site [4Fe-4S] cluster.

Serious consideration was given to the hypothesis that FumA catalyzes the fumarate-to-L-malate reaction during aerobic growth, while FumB catalyzes the L-malate-to-fumarate reaction as it would take place in the reductive pathway from oxaloacetate to succinate during anaerobic fermentative growth, as proposed by Woods et al. [1], [9]. This idea is persistent in the literature [10], [11], [18] as it is consistent with the differential regulation of the two genes under anaerobic and aerobic conditions. However, the K_m_ values reported by Woods et al. appear to be incorrect. The mistakenly presumed difference in K_m_ led to the conclusion that FumA has a higher affinity for fumarate than FumB. This statement is clearly not true. The results reported here indicate that the kinetic parameters for FumA and FumB are very similar for both substrates. The equilibrium constant for fumarate hydration as determined from our data, *K_eq_* = 2.8±0.8 and 4±1, is in close agreement with *K_eq_* = 4.4 as determined by others under similar experimental conditions [19], [20]. On the other hand, the data of Woods et al. led to very different equilibrium constants for FumA (*K_eq_* = 8.2) and FumB (*K_eq_* = 0.44). Contrary to Woods et al., Flint already mentioned that the kinetic constants of FumA and FumB are similar, referring to unpublished data that unfortunately have never been published [5].

For D-tartrate dehydratase activity, significant differences in the kinetic parameters of FumA and FumB were observed (Table 1). Although the K_m_-values of both enzymes for this substrate were similar, the *V_max_* of FumB was 5-fold higher than that of FumA. *E. coli* can grow anaerobically on D-tartrate when glycerol is supplied as an electron donor. Growth on D-tartrate was found to be seriously impaired in the *fumB* disruption mutant [2]. Since it is known that FumB is more expressed under anaerobic conditions, it was unclear if the FumB was a more efficient enzyme than FumA, or just expressed to a higher level under these conditions. However, we find clearly that FumB is a more efficient D-tartrate dehydratase than FumA. L-tartrate and meso-tartrate are not converted by FumA and FumB due to the stereochemistry of the reaction catalyzed by these enzymes. Not only the position of the hydroxyl group, but also of the hydrogen on the C3 position is important. Therefore meso-tartrate is not a substrate, while D-tartrate is. The same stereospecificity has also been found for the type II fumarases such as *E. coli* FumC and for the *P. furiosus* fumarase [5], [7].

The kinetic parameters for fumarase activity as determined in this study do not provide an explanation for the differential regulation of FumA and FumB for growth on rich media. Since FumA is expressed under aerobic conditions and FumB during anaerobic growth, a higher sensitivity towards oxygen of FumB as compared to FumA might be anticipated. However, the experiments performed in the present study did not reveal any difference in the oxygen sensitivity of the enzymes to substantiate this concept (Figure 4). FumA and FumB are known to be sensitive to oxidative stress as oxidation of the [4Fe-4S]^2+^ clusters causes the cluster to be unstable and lose the catalytic iron resulting in a [3Fe-4S]^+^ cluster [21]. Prolonged oxidative stress appears to result in further damage of the cluster to a state that cannot be regenerated.

The reduction potential E_m_ of −280 to −300 mV determined here for the [4Fe-4S] clusters in FumA and FumB is very different (i.e. circa 200 mV less negative) from the potential of approximately −500 mV reported for beef heart mitochondrial aconitase, another Lewis acid [4Fe-4S] enzyme [22], and from the potential of circa −480 mV previously reported for *E. coli* fumarase A [3]. This apparent discrepancy is explained as follows. The cluster in these non-redox, Lewis acid catalytic enzymes can exist in two, very different states: free versus substrate/product-bound. In the reduced state these two forms have different EPR spectra [3], [22] and, furthermore, the reduction potential of the free state is much lower than that of the bound state. The potential reported for aconitase is for the free state. In our hands, also the free state of the cluster in FumA and FumB is very hard to reduce: addition of excess dithionite affords only a barely detectable signal. Remarkably, a value of E_m,8_ = −480 mV was found by Flint et al. [3] for *E. coli* FumA with its cluster in the bound state. However, presumably in anticipation of a very low midpoint potential value, the redox titration was done in the presence only of low potential mediators (methyl viologen with E_m_ = −440 mV and triquat with E_m_ = −540 mV). Obviously, under these conditions a protein with a significantly less negative midpoint potential would fail to equilibrate with the platinum electrode, and the reported E_m_ = −480 mV is likely to be erroneous. We have repeated the titration as performed by Flint and coworkers on purified FumB (Figure S4), which showed scattered data points indicating that a redox equilibrium could not be attained during the experiment.

The fact that the reduction potential of the fumarate/L-malate bound [4Fe-4S] cluster is much higher than for the free [4Fe-4S] cluster may suggest that the [4Fe-4S]^1+^ cluster is stabilized upon substrate binding, suggesting that the affinity of the [4Fe-4S] cluster for the substrate is higher in the reduced (inactive) state than in the oxidized (active state). Perhaps tight binding of the substrate to the [4Fe-4S]^2+^ cluster has an adverse effect on the catalytic activity of the enzyme. In the present study it was found that in the presence of substrate/product the reduction potentials of the [4Fe-4S] cluster of FumA and that of FumB at pH 8.0 are not significantly different, i.e. approximately −290 mV. Since the intracellular redox potential of *E. coli* during aerobic growth has been estimated to be in the range of −260 to −280 mV [23], the cluster of FumA and FumB will be partially reduced, and hence in an inactive state [3]. A lower activity of the [4Fe-4S]^1+^ cluster has also been reported for aconitase, which retains 30% of its activity upon reduction [22]. This might be of physiological relevance as it may offer the cell another way to regulate enzyme activity.

In conclusion, the only significant biochemical difference between FumA and FumB that we were able to find is the rate of dehydration of D-tartrate. *E. coli* is able to grow anaerobically on D-tartrate if glycerol is supplemented as electron donor [2]. The metabolism of D-tartrate has been found to be dependent on DcuB and FumB. FumB has been proposed to catalyse the dehydration of D-tartrate and L-malate as part of the pathway for the conversion of D-tartrate to succinate. The results presented here show that FumB is more suitable for this role than FumA. The catalytic efficiency of FumB for the dehydration of L-malate is two orders of magnitude higher than of D-tartrate. Therefore, the physiological relevance of this biochemical difference between FumA and FumB remains arguable.

## Materials and Methods

### Enzyme Expression and Purification

The plasmids pGS57 and pGS56 containing *E. coli* FumA and B [24] were a gift from Dr. John Guest (Department of Microbiology, Sheffield University). The genes were cloned into a pET15b vector (Novagen) for N-terminal His-tag fusion protein production using standard techniques. The plasmids pET15b-FumA/B were transformed into *E. coli* BL21(DE3) cells. Cultures were grown in LB medium containing 100 µg/ml ampicillin at 37°C and 150 rpm. When the OD_600_ = 0.5 the temperature was lowered to 25°C, and protein expression was induced by the addition of 250 µM isopropyl-β-D-thiogalactoside. FumA and FumB without His-tags were produced as follows. The coding genes *fumA* and *fumB* were cloned into pET9a with the NdeI and BamHI fragment derived from pET15b-*fumA* and pET15b-*fumB*. *E. coli* BL21(DE3)pLysS was transformed with pET9a-*fumA* and pET9a-*fumB*. Precultures were grown in LB medium containing 50 µg/ml Kanamycin overnight at 37°C and 150 rpm, diluted 100 fold and was induced at 25°C by the addition of 200 µM isopropyl-β-D-thiogalactoside.

After 16 hours expression, cells were harvested by centrifugation. Cell pellets were resuspended in 50 mM Tris buffer pH 8, supplemented with 5% glycerol, 0.5 mM dithiothreitol (DTT), 1 mM phenylmethylsulfonylfluoride and 10 mg lysozyme and DNase. Cells were disrupted in a cell disrupter (Constant systems). The lysate was flushed with argon and transferred to an anaerobic box for further purification of the his-tagged enzymes on a Nickel sepharose column. The purification of the proteins without his-tag was performed with an Akta purifier (GE Healthcare) using a DEAE sepharose column and a linear gradient of 0 to 500 mM KCl in 50 mM Tris buffer pH 8, supplemented with 5% glycerol, 0.5 mM dithiothreitol (DTT). Fractions were analysed on SDS PAGE and the brown fractions were concentrated (15×) using Amicon Ultra-15 centrifugal filter units (Millipore).

Protein purity and molecular weight were assessed with SDS PAGE, and protein concentrations were determined using the bicinchoninic acid assay. Size exclusion chromatography (Superdex 200) was used to estimate the molecular weight of the native enzyme. FumA and FumB without His-tags were only used for the redox titrations.

### EPR Spectroscopy

EPR tubes containing 200 µl purified enzyme ‘as isolated’ were frozen in liquid nitrogen to study the [3Fe-4S]^+^ cluster present after nickel column purification. To study the [4Fe-4S]^1+^ cluster, purified enzyme was incubated for 60 minutes at 0°C with 0.5 mM Fe^2+^ and 0.25 mM L-cysteine to regenerate the [3Fe-4S] cluster to [4Fe-4S] [3]. Subsequently, the samples containing 0.25 mM His-tagged fumarase were reduced with 10 mM sodium dithionite, 5 mM fumarate was added, and 200 µl aliquots were frozen in EPR tubes. EPR data were recorded on a Bruker ER200D EPR spectrometer with a National Instruments interface and in house developed mixed-language data acquisition and analysis software written in LabVIEW and FORTRAN 90/95.

### Iron Concentration Determination

To estimate cluster content of the enzyme, the iron concentration was determined using the ferene method as described previously [25]. The determination was performed in triplicate using 3 nmol purified enzyme, with or without prior regeneration of the cluster. A BioSpin column (Biorad) was used to remove excess iron.

### Redox Titration

To determine the midpoint potential of the [4Fe-4S] clusters of FumA and FumB, EPR monitored redox titrations were performed in an anaerobic glovebox (Coy) on purified enzyme without his-tag. The titration buffer was 100 mM 3-[4(2-Hydroxyethyl)-1-piperazinyl] propanesulfonic acid (EPPS) buffer pH 8 with 5% glycerol and 250 mM NaCl. A mediator mix (160 µM of each mediator) was prepared in the same buffer containing per 100 ml 5.2 mg dichlorophenol indophenol, 5.4 mg phenazine ethosulfate, 6 mg methylene blue, 3.8 mg resorufine, 7.5 mg indigodisulfonate, 2.8 mg 2-OH-1,4-naphtaquinone, 5.3 mg anthraquinone-2-sulfonate, 5.2 mg phenosafranin, 5.6 mg safranin O, 4.6 mg neutral red, 6.6 mg benzyl viologen and 5 mg methyl viologen. To the titration vessel 0.15 mM FumA or FumB, 80 µM mediator mix and 5 mM fumarate was added in a total volume of 2.2 ml. The titration cell was connected to a reference Ag/AgCl electrode (SSE) and a platinum wire electrode. The electrodes were connected to a voltmeter. The potential was varied using sodium dithionite and potassium ferricyanide as reductant and oxidant, respectively. At various potentials, samples of 200 µl were taken from the titration vessel, transferred to an EPR tube, and immediately frozen in liquid nitrogen. EPR spectra were recorded as described above.

### Enzyme Activity

Fumarase activity of FumA and FumB was assayed inside an anaerobic glove box by monitoring fumarate formation or depletion at 250 nm. Assays were performed at 37°C in assay buffer (100 mM EPPS buffer pH 8 with 5% glycerol and 0.5 mM DTT) supplemented with various substrate concentrations, and initiated by 0.1 µM regenerated enzyme. The regeneration procedure was as described above in the EPR spectroscopy paragraph.

Tartrate dehydratase activity was determined in an anaerobic glovebox using a coupled assay [2] in which the oxaloacetate formed by FumA/B is converted by the NADH-dependent enzyme malate dehydrogenase. The concomitant NADH depletion was monitored at 340 nm. The reaction was carried out by 0.4 µM regenerated fumarase at 37°C in assay buffer with 100 µM NADH, 1 mM MgCl_2_, 0.006 U L-malate dehydrogenase (Sigma) and varying concentrations of D-tartrate, L-tartrate or meso-tartrate.

The obtained kinetic data were fitted to the Michaelis-Menten equation using the data analysis software Igor (Wavemetrics). The reported error-values of the kinetic parameters are one standard deviation of the fit.

### Stability of FumA and FumB

To determine the stability of FumA and FumB towards inactivation by molecular oxygen, the clusters were first regenerated in the anaerobic glovebox, and fumarase activity was measured under anaerobic conditions. Next, the enzymes were removed from the box in airtight containers. The samples were 100-fold diluted in air saturated buffer (100 mM EPPS buffer pH 8 with 5% glycerol) and incubated for 0, 0.5, 1 or 2 minutes, and subsequently the residual enzyme activity was determined after the addition of 0.5 mM fumarate.

To determine the effect of reduction of the [4Fe-4S]^2+^ cluster on fumarase activity, FumA and FumB were photoreduced by irradiation with a Xenon lamp for 10 minutes in the presence of 5 µM proflavin, similar to what has been described for FumA [26]. After photoreduction, residual fumarase activity was measured. The clusters were re-oxidized by the addition of stoichiometric amounts of thionine or phenazine ethosulfate.

## Supporting Information

Figure S1 **SDS-PAGE of** ***E. coli*** **pET15b-fuma and** ***E. coli*** **pET15b-fumb after induction.** The arrow marks the position of Fumarase A and B. Lane 1: 10 µl *E. coli* pET15b-fuma before induction; lane 2: 30 µl *E. coli* pET15b-fuma after induction; lane 3: 10 µl *E. coli* pET15b-fuma after induction; lane 4: 30 µl *E. coli* pET15b-fumb after induction; lane 4: 10 µl *E. coli* pET15b-fumb after induction.
(TIF)

Figure S2 **EPR spectrum of lysed cells of** ***E. coli*** **containing pET15b-** ***fumB*** **overexpressing FumB after reduction with 5 mM sodium dithionite and addition of 5 mM L-malate.** EPR parameters: microwave frequency 9.407 GHz; microwave power 20 mW; modulation frequency 100 kHz; modulation amplitude 1.25 mT; temperature 13.8 K.
(TIF)

Figure S3 **Michaelis-Menten kinetics of** ***E. coli*** **FumA and FumB.** a fumarate hydration. b L-malate dehydration. c D-tartrate dehydration by *E. coli* fumA and FumB. Assays were performed anaerobically at 37°C in assay buffer (100 mM EPPS buffer pH 8 with 5% glycerol and 0.5 mM DTT), varying concentrations of fumarate or L-malate and initiated by 0.1 µM regenerated enzyme. Tartrate dehydratase activity was determined in a coupled assay [2] with 0.4 µM regenerated fumarase at 37°C in assay buffer with 100 µM NADH, 1 mM MgCl_2_, 0.006 U L-malate dehydrogenase and varying concentrations of D-tartrate.
(TIF)

Figure S4 **Redox titration on purified FumB as performed by the method of Flint and coworkers** [**3**] **.** *Upper panel* Nernst plot of the g_y_-signal amplitude versus the redox potential. *Lower panel* EPR spectra of the redox titration samples.
(TIF)

Table S1 **Original data used to obtain the kinetic parameters of the fumarase and tartrate dehydratase activity of** ***E. coli*** **FumA and FumB.**
(DOCX)
