# Supplementary figures and images for: Biochemical Similarities and Differences between the Catalytic [4Fe-4S] Cluster Containing Fumarases FumA and FumB from Escherichia coli
Source: PLoS One. 2013 Feb 6;8(2):e55549. doi: 10.1371/journal.pone.0055549 (PMC3565967; doi:10.1371/journal.pone.0055549)

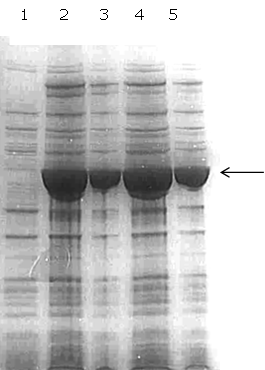

Supplement: Figure S1 — SDS-PAGE of E. coli pET15b-fuma and E. coli pET15b-fumb after induction. The arrow marks the position of Fumarase A and B. Lane 1: 10 µl E. coli pET15b-fuma before induction; lane 2: 30 µl E. coli pET15b-fuma after induction; lane 3: 10 µl E. coli pET15b-fuma after induction; lane 4: 30 µl E. coli pET15b-fumb after induction; lane 4: 10 µl E. coli pET15b-fumb after induction. (TIF) [file pone.0055549.s001.tif]

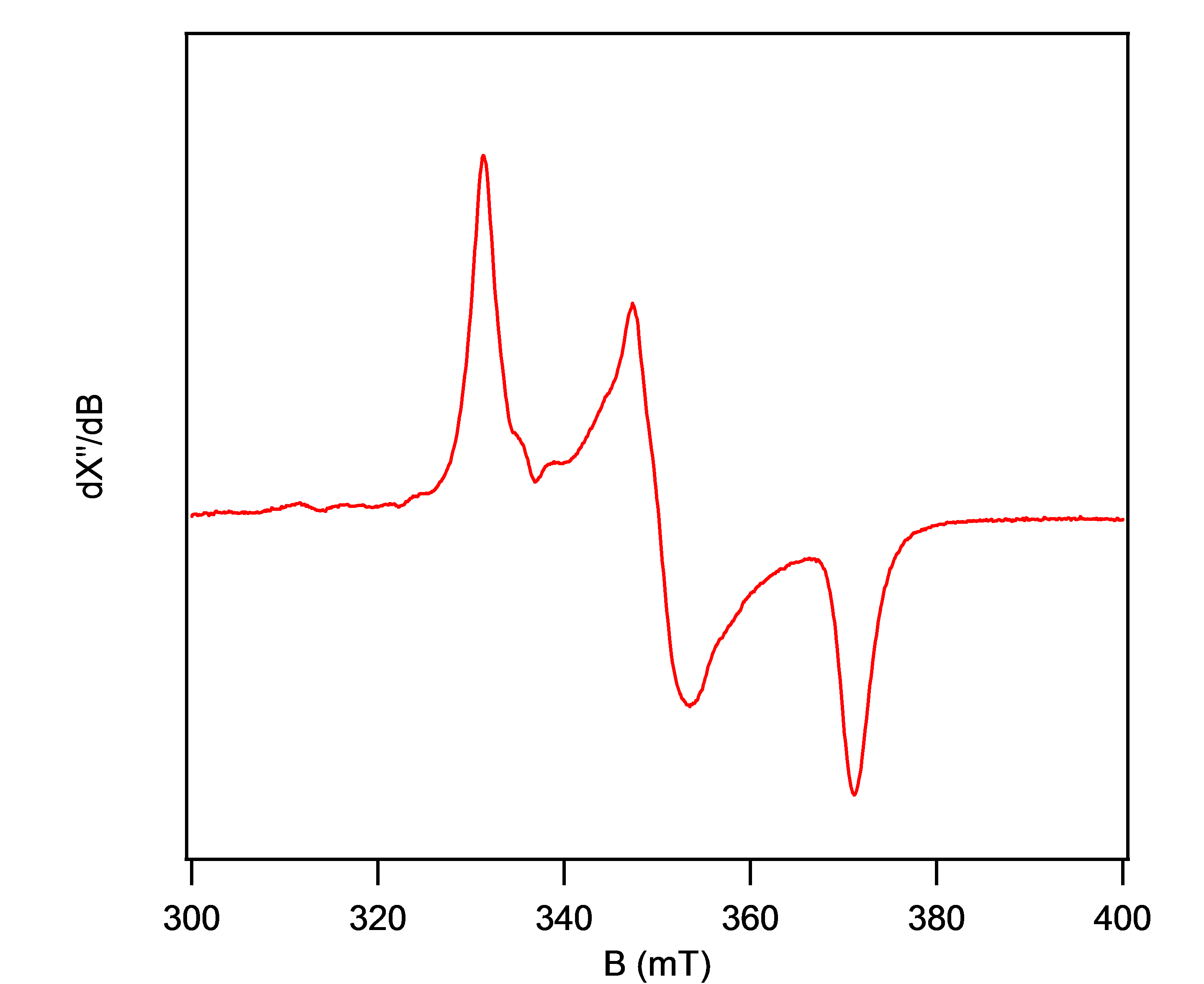

Supplement: Figure S2 — EPR spectrum of lysed cells of E. coli containing pET15b- fumB overexpressing FumB after reduction with 5 mM sodium dithionite and addition of 5 mM L-malate. EPR parameters: microwave frequency 9.407 GHz; microwave power 20 mW; modulation frequency 100 kHz; modulation amplitude 1.25 mT; temperature 13.8 K. (TIF) [file pone.0055549.s002.tif]

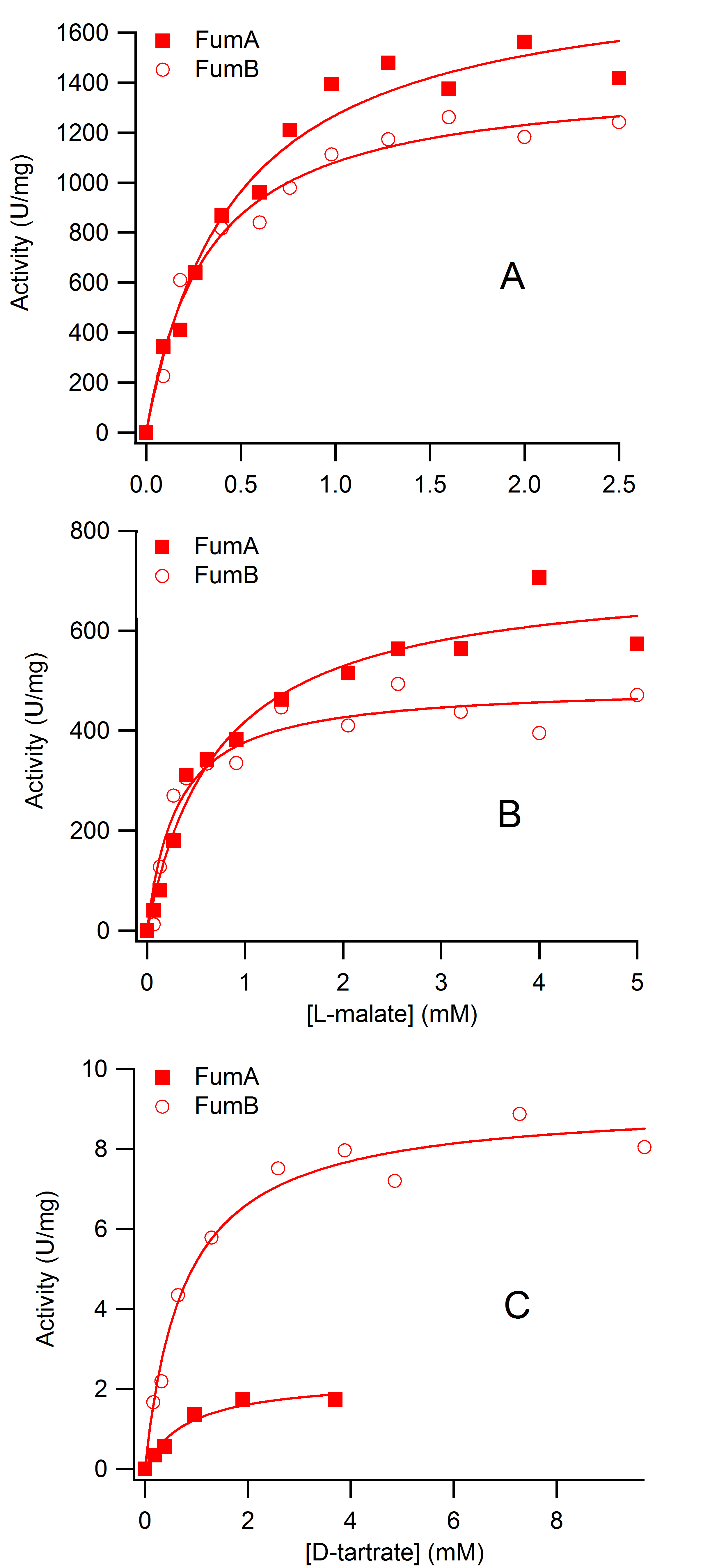

Supplement: Figure S3 — Michaelis-Menten kinetics of E. coli FumA and FumB. a fumarate hydration. b L-malate dehydration. c D-tartrate dehydration by E. coli fumA and FumB. Assays were performed anaerobically at 37°C in assay buffer (100 mM EPPS buffer pH 8 with 5% glycerol and 0.5 mM DTT), varying concentrations of fumarate or L-malate and initiated by 0.1 µM regenerated enzyme. Tartrate dehydratase activity was determined in a coupled assay [2] with 0.4 µM regenerated fumarase at 37°C in assay buffer with 100 µM NADH, 1 mM MgCl2, 0.006 U L-malate dehydrogenase and varying concentrations of D-tartrate. (TIF) [file pone.0055549.s003.tif]

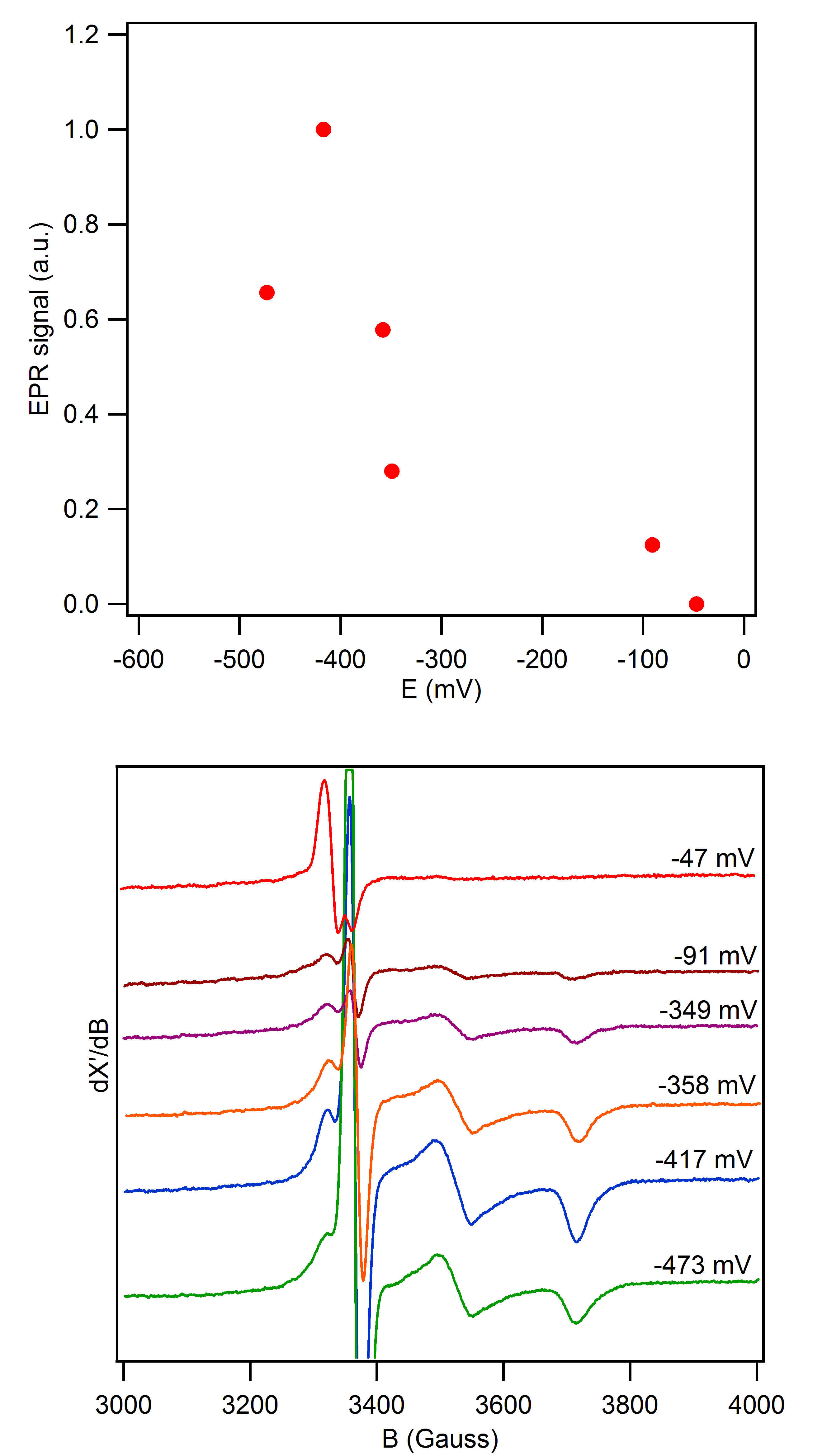

Supplement: Figure S4 — Redox titration on purified FumB as performed by the method of Flint and coworkers [3] . Upper panel Nernst plot of the gy-signal amplitude versus the redox potential. Lower panel EPR spectra of the redox titration samples. (TIF) [file pone.0055549.s004.tif]
