# Supplementary material for: Biochemical Similarities and Differences between the Catalytic [4Fe-4S] Cluster Containing Fumarases FumA and FumB from Escherichia coli
Source: PLoS One. 2013 Feb 6;8(2):e55549. doi: 10.1371/journal.pone.0055549 (PMC3565967; doi:10.1371/journal.pone.0055549)
Supplement: Table S1 — Original data used to obtain the kinetic parameters of the fumarase and tartrate dehydratase activity of E. coli FumA and FumB. (DOCX) [file pone.0055549.s005.docx]

**Table S1.** Original data used to obtain the kinetic parameters of the fumarase and D-tartrate dehydratase activity of *E. coli* FumA and FumB

| **fumarate🡪L-malate** | | | **L-malate 🡪 fumarate** | | | **D-tartrate 🡪 oxaloacetate** | | |
| --- | --- | --- | --- | --- | --- | --- | --- | --- |
| **[fumarate] (mM)** | **FumA activity (U/mg)** | **FumB activity (U/mg)** | **[L-malate] (mM)** | **FumA activity (U/mg)** | **FumB activity (U/mg)** | **[D-tartrate (mM)** | **FumA activity (U/mg)** | **FumB activity (U/mg)** |
| 0.09 | 344 | 226 | 0.067 | 40.6 | 12 | 0.19 | 0.34 |  |
| 0.18 | 411 | 609 | 0.13 | 80.9 | 128 | 0.38 | 0.56 |  |
| 0.26 | 640 | 638 | 0.27 | 180 | 270 | 0.96 | 1.36 |  |
| 0.4 | 868 | 817 | 0.4 | 312 | 304 | 1.9 | 1.73 |  |
| 0.6 | 961 | 840 | 0.61 | 342 | 334 | 3.7 | 1.73 |  |
| 0.76 | 1210 | 979 | 0.91 | 382 | 335 | 0.16 |  | 1.66 |
| 0.98 | 1394 | 1112 | 1.37 | 463 | 446 | 0.32 |  | 2.19 |
| 1.28 | 1479 | 1173 | 2.05 | 516 | 410 | 1.29 |  | 4.34 |
| 1.6 | 1375 | 1261 | 2.56 | 563 | 494 | 2.59 |  | 5.78 |
| 2 | 1562 | 1183 | 3.2 | 565 | 438 | 3.88 |  | 7.52 |
| 2.5 | 1419 | 1242 | 4 | 706 | 395 | 4.85 |  | 7.96 |
|  |  |  | 5 | 574 | 471 | 7.28 |  | 7.20 |
|  |  |  |  |  |  | 9.71 |  | 8.87 |
|  |  |  |  |  |  |  |  | 8.04 |
